# Medical Education in East Asia: Past and Future

**DOI:** 10.5195/jmla.2019.639

**Published:** 2019-04-01

**Authors:** Gregg A. Stevens

**Affiliations:** Health Sciences Library, Stony Brook University, Stony Brook, NY, gregg.stevens@stonybrook.edu

In today’s highly connected world, when information sweeps across the globe in minutes, we can sometimes forget how the power of cultural influence is not just limited to the age of the Internet. Over the past several centuries, explorers, missionaries, and merchants from Europe and North America have brought many elements of Western culture—for better or worse—to other parts of the globe. Among their exports were Western medicine and the various modes of Western medical education.

This collection of essays outlines the history of medical education in five East Asian countries and territories: China, Japan, South Korea, Taiwan, and Hong Kong. Medical schools in all five countries received funding from the China Medical Board (CMB), a philanthropic organization originating from the Rockefeller Foundation. These funds were used to establish and maintain Western-style hospitals, schools, and support services throughout the twentieth century. CMB funding was critical to improving health care in many of these countries, especially in the wake of World War II, when national infrastructures were destroyed and in dire need of replacement. Medical libraries were specifically noted as recipients of CMB funding in the post-war years. Funds were used to build up book and journal collections across the region, including the only medical library in the Philippines, illustrating the important role that libraries have played in medical education.

The history of the CMB is the first essay in this collection, followed by an essay on the role that medical education and cooperation played in US foreign policy in East Asia during the mid-twentieth century. The heart of the collection, though, is a series of five essays describing the development of the medical education system in each country, from the beginnings of Western-style education in each area to the current medical challenges that each nation faces.

Despite the differences in medical education systems, many common themes emerge from the essays. All five countries transitioned away from their traditional medical models because of various Western influences. China’s medical education system changed throughout the twentieth century, as European, Japanese, and Soviet models came and went in the Chinese schools. Japan used a German-style educational model for many years, until compelled to adopt the US model after World War II. The US style of education was also used in South Korea and Taiwan, while the British colony of Hong Kong adapted the system used in the United Kingdom. However, traditional medicine still exists throughout East Asia, with parallel schools and clinics.

Western medicine and medical education may be the dominant system now, but they have by no means completely replaced local traditions. As one essay author noted, “in the face of the globalization of medical education, we must not unreflectively assume that educational practices in the West are the best. While maintaining awareness of best practices globally, we also have to develop a curriculum that respects local cultural values” (p. 104).

With populations that are growing, aging, and suffering less from the burden of infectious disease, these countries have a common concern that not enough physicians are being trained. The doctors who are completing their medical education are gravitating toward urban areas and medical specialization, leaving critical gaps in primary care and in rural areas. The shortages of primary care physicians in less populated areas may sound familiar to those who work in medical education in North America, exemplifying that the inherent qualities of medical education and health care are truly global.

The essays are well written, and many contain tables of statistical information on the medical schools in each country. The collection can serve as a good reference source for anyone researching Asian medicine or medical education, as well as those researching the impact of modern Western medicine. The book is recommended for academic health sciences libraries, especially those with collections in the history of health sciences or in global health.

